# Antibiotic Use Patterns at Jimma Medical Center in Southwest Ethiopia: A Call for Local Antibiogram-Guided Prescription

**DOI:** 10.3390/jcm14072413

**Published:** 2025-04-01

**Authors:** Mulatu Gashaw, Melkamu Berhane, Sisay Bekele, Tsegaye Melaku, Gemechu Lemmi, Legese Chelkeba, Tekle Wakjira, Getnet Tesfaw, Zeleke Mekonnen, Arne Kroidl, Andreas Wieser, Guenter Froeschl, Thomas Seeholzer, Solomon Ali, Esayas Kebede Gudina

**Affiliations:** 1 School of Medical Laboratory Science, Jimma University, Jimma P.O. Box 378, Ethiopia; 2 CIHLMU Center for International Health, University Hospital, LMU Munich, Leopoldstrasse 5, 80802 Munich, Germany; 3 Department of Pediatrics and Child Health, Jimma University, Jimma P.O. Box 378, Ethiopia; 4 Department of Ophthalmology, Jimma University, Jimma P.O. Box 378, Ethiopia; 5 School of Pharmacy, Jimma University, Jimma P.O. Box 378, Ethiopia; 6 Department of Surgery, Jimma University, Jimma P.O. Box 378, Ethiopia; 7 Department of Pharmacology and Clinical Pharmacy, College of Health Sciences, Addis Ababa University, Addis Ababa P.O. Box 1176, Ethiopia; 8 Department of Gynecology and Obstetrics, Jimma University, Jimma P.O. Box 378, Ethiopia; 9 German Center for Infection Research (DZIF), Partner Site Munich, 80337 Munich, Germany; 10 Institute of Infectious Diseases and Tropical Medicine, University Hospital Ludwig, Maximilians-Universität, 80539 Munich, Germany; 11 Max von Pettenkofer-Institute (Medical Microbiology), LMU Munich, 80539 Munich, Germany; 12 Fraunhofer Institute for Translational Medicine and Pharmacology ITMP, Immunology, Infection and Pandemic Research IIP, Türkenstraße 87, 80799 Munich, Germany; 13 Department of Microbiology, Parasitology, and Immunology, St. Paul’s Hospital Millennium Medical College, Addis Ababa P.O. Box 1271, Ethiopia; 14 Department of Internal Medicine, Jimma University, Jimma P.O. Box 378, Ethiopia

**Keywords:** antimicrobial resistance, antimicrobial stewardship, empiric antibiotic use, ceftriaxone, diagnostic stewardship, Ethiopia

## Abstract

**Background:** The discovery of antibiotics revolutionized healthcare by significantly reducing morbidity and mortality. However, excessive and inappropriate use has led to a global surge in antimicrobial resistance, particularly in low- and middle-income countries. This study aimed to evaluate antibiotic use patterns among inpatients at Jimma Medical Center (JMC) in Southwest Ethiopia. **Methods:** A longitudinal observational study was conducted in February and March 2019 at JMC, focusing on patients admitted for over 24 h who received antibiotics. Data on patient demographics, clinical indications, and antibiotics prescribed were systematically collected. Antibiotic consumption rates were measured as days of therapy (DOTs) per 100 patient-days, and utilization was classified according to the World Health Organization (WHO) AWaRe (Access, Watch, and Reserve) framework. **Results:** A total of 384 inpatients were included, with a male predominance (53.9%) and a median age of 24 years (IQR: 5–37). In total, 634 antibiotic regimens were prescribed. According to the WHO AWaRe classification, 48.3% (306/634) were “Access” and 51.7% (328/634) were “Watch” antibiotics. Patients were treated with antibiotics for a median duration of 4 days (IQR: 2–7), leading to a total of 2880 days of antibiotic therapy. Ceftriaxone was the most commonly prescribed antibiotic, with a usage rate of 44.65 DOTs per 100 patient-days. Substantial variability was observed in empirical antibiotic regimens among treating physicians and across wards. Culture and antibiotic susceptibility testing (AST) were performed for only 4.2% of patients, and none of the treatments were modified based on susceptibility data. **Conclusions:** The study highlights critical issues in antibiotic prescribing at JMC, including over-reliance on “Watch” antibiotics, predominantly ceftriaxone, limited use of AST results, and deviations from standard treatment guidelines. Addressing these challenges requires implementing antimicrobial stewardship programs, developing evidence-based local treatment guidelines, and strengthening and encouraging the use of microbiology services to improve rational antibiotic use.

## 1. Introduction

The discovery of antibiotics has been one of the most impactful advancements in modern medicine, drastically reducing the burden of infectious diseases globally. Antibiotics have become essential in treating bacterial infections, facilitating surgical procedures, and supporting complex medical care [1,2]. However, the overuse and misuse of antibiotics have led to the widespread emergence of antimicrobial resistance (AMR), compromising the efficacy of these life-saving drugs [3,4,5]. Inappropriate antibiotic use contributes to AMR and exerts pressure for the selection of bacterial species resistant to various classes of antibiotics, further exacerbating the AMR crisis [6].

The surge of antibiotic resistance poses a significant threat, as bacterial infections that were once easily treatable are now becoming resistant to available antibiotics [3]. AMR is recognized as a global concern, leading to increased healthcare costs and emerging as a significant cause of mortality, particularly in low-income settings [7,8]. Despite the urgent need, the development of new antibiotics has not kept pace with the rapid evolution of AMR, intensifying the problem [3].

In low- and middle-income countries (LMICs), the challenge of AMR is exacerbated by inadequate regulatory frameworks [4,9,10], poor adherence to treatment guidelines [11], and suboptimal quality control measures [12], all of which contribute to irrational antibiotic use. In these countries, 70–90% of consumed antibiotics are imported without sufficient quality control, and over 20% of these are estimated to be counterfeit [12,13].

In Ethiopia, the over-prescription of broad-spectrum antibiotics is a significant concern, primarily due to limited diagnostic capacity, which often necessitates empirical treatment practices [14,15]. This issue is particularly pronounced in hospitals where diagnostic tools may not be readily available, forcing clinicians to prescribe broad-spectrum antibiotics as a precautionary measure to cover a range of possible infectious etiologies [16]. This over-reliance on broad-spectrum antibiotics has been observed across several LMICs, including Ethiopia, exacerbating the problem of AMR [17].

Ethiopia faces several key challenges in its fight against AMR. Firstly, there is limited implementation of antimicrobial stewardship (AMS), leading to widespread misuse and overuse of antibiotics [18]. The surveillance system for tracking resistance patterns is also inadequate, hampered by infrastructure gaps and inconsistent data sharing across regions. Additionally, resource constraints limit funding for diagnostic improvements, awareness campaigns, and public health initiatives [19].

This study aimed to evaluate inpatient antibiotic use patterns at Jimma Medical Center (JMC), providing insights to guide the development of local antibiogram-based treatment protocols. Optimizing antibiotic therapy through such targeted approaches is essential for mitigating the growing threat of AMR.

## 2. Methods

### 2.1. Study Design, Period, and Setting

A longitudinal observational study was conducted at JMC, an 800-bed tertiary care hospital in Southwest Ethiopia, in February and March 2019. The center provides healthcare services in a catchment area with over 20 million inhabitants and has more than 20,000 inpatient admissions each year. This study covered patients admitted to all wards of JMC, including internal medicine, pediatrics, surgery, obstetrics and gynecology, and intensive care units.

### 2.2. Study Participants

Patients of all age groups who were admitted to JMC for more than 24 h and received antibiotics for therapeutic or prophylactic reasons were included. Patients receiving only antituberculosis treatment were excluded unless additional antibiotics were used.

The sample size was determined using the single population proportion formula for the observational longitudinal study. In the absence of prior studies conducted in the setting, a prevalence of antibiotic use of 50% was assumed. With a 95% confidence interval and a 0.05 margin of error taken into consideration, this resulted in a total sample size of 384. All admitted patients receiving antibiotics were consecutively recruited until the calculated sample size was reached.

### 2.3. Data Collection

Baseline data on demographics, clinical indications, and comorbidities were collected through patient interviews and medical record reviews. Details on the type, dose, duration, and route of antibiotics were obtained via daily record reviews. Antibiotic consumption rates were calculated using days of therapy (DOTs) per 100 patient-days.

In order to assess optimal use of antibiotics, the antibiotics were classified into “Access”, “Watch”, or “Reserve” (AWaRe) categories based on the 2019 World Health Organization (WHO) AWaRe classification. Access antibiotics are first- and second-line options for common infections and are readily available in all countries and facilities. Examples include amoxicillin, amoxicillin-clavulanic acid, ampicillin, cefalexin, chloramphenicol, cloxacillin, trimethoprim-sulfamethoxazole, doxycycline, gentamicin, metronidazole, and penicillin. The Watch group includes antibiotic classes that should be prescribed only for specific indications due to their higher potential for resistance. This group includes azithromycin, ceftriaxone, ciprofloxacin, clarithromycin, norfloxacin, ceftazidime, and vancomycin. Reserve group antibiotics are reserved for treatment of confirmed or suspected infections due to multidrug-resistant organisms; they are considered “last resort” options [20].

### 2.4. Data Analysis

The data were entered into EpiData version 4.2 and subsequently transferred to SPSS version 26, Microsoft Excel 2016, and GraphPad Prism version 8.4.3 for analysis. Antibiotic use is presented in frequency distributions and proportions and displayed using tables and graphs. In addition, the crude antibiotic consumption rate was calculated using days of therapy (DOTs) per 100 patient-days, defined as at least one dose of a selected antibiotic given on a calendar day. A patient on multiple antibiotics on the selected list was counted for each separate antibiotic given on each calendar day.

### 2.5. Ethical Considerations

Ethics approval was obtained from the institutional review board of Jimma University Institute of Health (reference number: IHRPGO/495/2018) and the Ethics Committee of the Medical Faculty of Ludwig-Maximilians-Universität of Munich, Germany (Project No: 21-0157). Written informed consent was obtained from the patients, or from parents or caregivers for children and severely ill or unconscious patients. Confidentiality of the data was ensured through anonymization and storage of data in password-protected computers. The study adhered to the principles outlined in the most recent version of the Declaration of Helsinki.

## 3. Results

### 3.1. Patient Demographics and Clinical Characteristics

A total of 384 patients participated in the study, with a median age of 24 years (IQR: 5–37 years). Among the participants, 53.9% were male, and most (65.1%) lived in rural areas. About a quarter of the participants were from the pediatric ward, and nearly 16% of the participants were recruited from the emergency department. Infectious diseases were the main reason for admission in 45.0% of the patients. The most common reasons for these admissions included skin and soft tissue infections (9.9%), pneumonia (8.3%), sepsis (8.1%), and bacterial meningitis (6.3%). Underlying chronic medical conditions were reported in 12.0% of patients, with diabetes mellitus (3.1%) and hypertension (3.1%) being the most common (Table 1).

### 3.2. Indication for Antibiotics

Over two-thirds (70.3%, 270/384) of participants received antibiotics for therapeutic reasons. However, only 45% (173/384) of these individuals were admitted primarily due to infectious diseases (Table 1). For the remaining 25.3% (97/384) of patients, antibiotics were prescribed to treat healthcare-associated infections or secondary infections that were not the main reasons for their hospital admission (Table 2). On the other hand, 29.7% (114/384) of participants received antibiotics for prophylactic purposes. Among these, the majority of them (83.3%, 95/114) were given perioperative prophylaxis to prevent infections during surgical procedures (Table 2).

### 3.3. Antibiotic Regimen and Prescription Patterns

**Figure 1:**
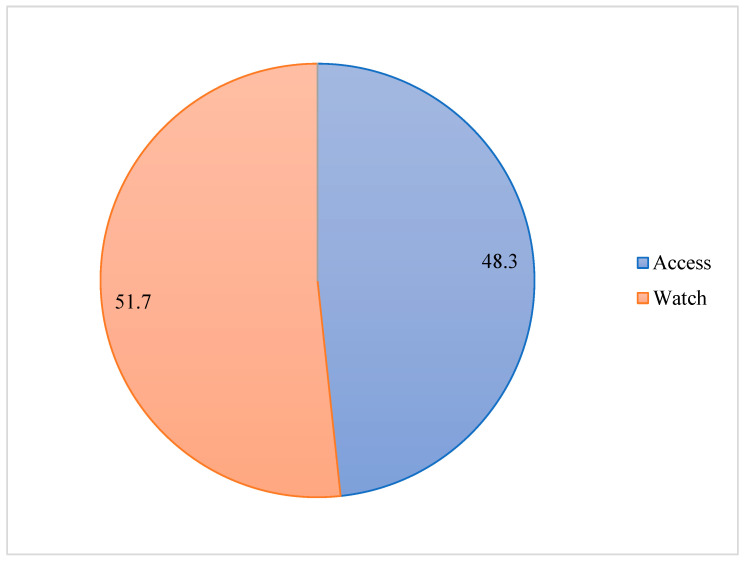
Proportion of prescribed antibiotics according to WHO AWaRe classification.

**Figure 2:**
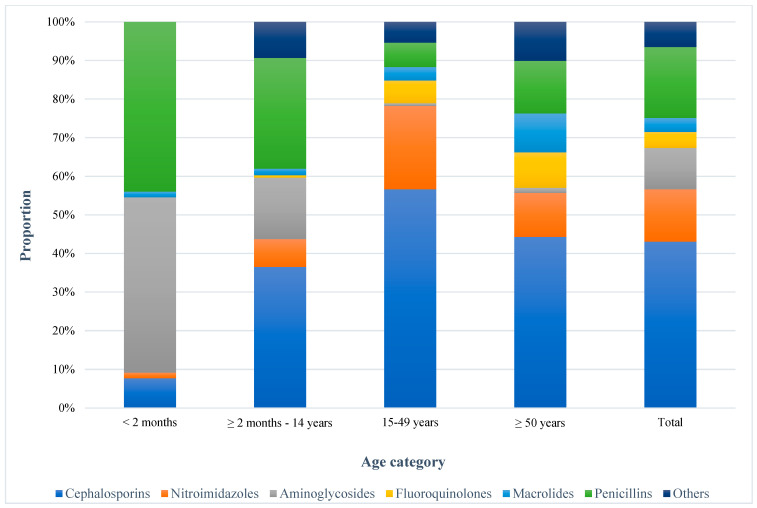
Frequency of administered antibiotic classes for inpatient care. Others: chloramphenicol + Glycopeptides + Tetracyclines + trimethoprim-sulfamethoxazole.

During the study period, 634 antibiotic regimes were prescribed for 384 participants, encompassing both single and combined antibiotic regimens. According to the WHO AWaRe classification, 48.3% (306/634) of the antibiotics were “Access” groups: amoxicillin (12), amoxicillin-clavulanic acid (23), ampicillin (54), cefalexin (4), chloramphenicol (20), cloxacillin (21), trimethoprim-sulfamethoxazole (2), doxycycline (9), gentamicin (68), metronidazole (86), and penicillin (7). Meanwhile, 51.7% (328/634) belong to the “Watch” category: azithromycin (22), ceftriaxone (268), ciprofloxacin (23), clarithromycin (1), norfloxacin (3), ceftazidime (1), and vancomycin (10) (Figure 1). The distribution of antibiotic classes varied across different age groups. Cephalosporins were the most frequently prescribed antibiotic classes, used by 71.1% (273/384) of the patients, with the highest usage observed in the 15–49 years of age group at 42.2% (162/384), followed by penicillins (30.4%, 117/384), nitroimidazoles (22.4%, 86/384), and aminoglycosides (17.7%, 68/384). However, more than half of the aminoglycosides were prescribed to patients under two months of age. Fluoroquinolones (6.8%, 26/384) and macrolides (6.0%, 23/384) were prescribed less frequently (Figure 2). About 92.1% (105/114) of the patients who received antibiotics as prophylaxis and 60.0% (162/270) of those who received antibiotics for therapeutic purposes took ceftriaxone, either alone or in combination with other antibiotics.

**Figure 3:**
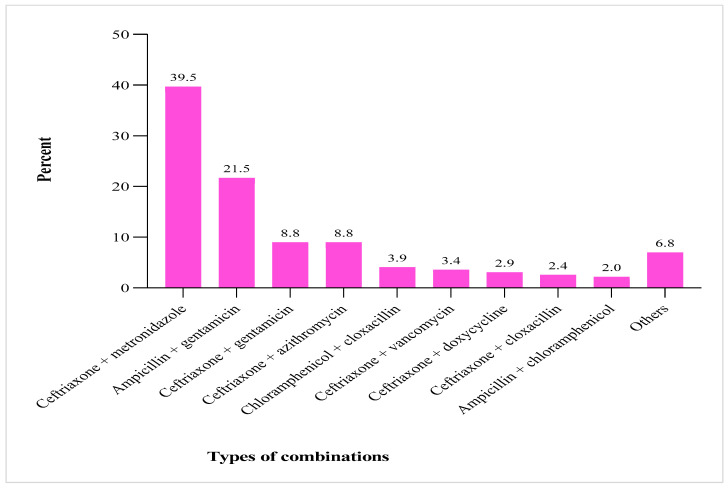
Frequency of administered antibiotic combination therapies. Others: ceftriaxone + vancomycin; ceftriaxone + ciprofloxacin; cephalexin + metronidazole; ceftazidime + vancomycin; gentamicin + vancomycin; amoxicillin + clarithromycin; and ceftriaxone + chloramphenicol.

In total, combination therapy was used in 53.4% (205/384) of patients, and 3.4% (13/384) received a triple regimen. The most frequently used combination was ceftriaxone with metronidazole (39.5%, 81/205), followed by ampicillin with gentamicin (21.5%, 44/205), ceftriaxone with gentamicin (8.8%, 18.205), and ceftriaxone with azithromycin (8.8%, 18/205) (Figure 3).

Days of Therapy (DOTs): Patients received antibiotic regimens for a median duration of 4 days (IQR: 2–7), resulting in a total of 2880 days of antibiotic therapy for 384 patients. For therapeutic regimens, the median duration of antibiotic use was 5 days (IQR: 3–7), ranging from 1 to 24 days. In contrast, the median duration for antibiotics used prophylactically was 3 days (IQR: 1–7), with a range of 1 to 17 days. Ceftriaxone was the most commonly used antibiotic, with a utilization rate of 44.65 DOTs per 100 patient-days, followed by metronidazole (16.67 DOTs/100 patient-days) and gentamicin (10.87 DOTs/patient-days) (Table 3).

Route of Administration: Intravenous (IV) administration was the predominant route, with 82.0% (520/634) of all antibiotic regimens given via this route (Table 4). Nearly all prophylactic regimens (98.5%, 130/132) and 78.9% (396/502) of therapeutic regimens were administered parenterally, either IV (520) or intramuscularly (6). Overall, 88.3% (339/384) of the patients received antibiotics intravenously.

### 3.4. Empiric Antibiotic Prescription Patterns and Guideline Compliance

The selection of initial empiric antibiotics for similar indications was different among treating physicians and wards of admissions. For instance, adult patients with severe community-acquired bacterial pneumonia without comorbidities were treated with three different options: (1) ceftriaxone alone, (2) ceftriaxone with azithromycin, or (3) ceftriaxone with doxycycline. Similarly, nine different regimens were used for the treatment of presumed community-acquired bacterial meningitis (Table 4). When evaluated against the 2014 Standard Treatment Guidelines by the Ethiopian Food and Drug Authority [21], empiric antibiotic use did not comply with this guideline for 73.4% (116/158) of pediatric and 38.1% (86/226) of adult patients. Additionally, none of the empirically prescribed antibiotics were supported by the antibiogram data observed in this study.

### 3.5. Diagnostic Utilization and Patient Outcomes

Culture and antibiotic susceptibility testing (AST) were performed for only 4.2% (16/384) of patients, including those with sepsis (8), meningitis (3), sexually transmitted infections (2), and urinary tract infections (1). Bacterial growth was reported in seven cases; however, none of these patients received adjusted antibiotic treatment based on the AST results. This limitation may have adversely affected patient outcomes, as only 6.0% (23/384) of patients had their initial empiric antibiotic therapy modified, with all changes relying solely on clinical judgment. More than six percent (24/384) of patients died, including 16 children under two months old who were admitted for hyaline membrane disease (7), sepsis (5), and meningitis (4). Additionally, 2.1% (8/384) of patients were referred for further care, and 2.9% (11/384) left the hospital against medical advice, whereas the outcomes of 2 patients were unknown.

## 4. Discussion

The findings from this study offer critical insights into antibiotic prescribing practices, diagnostic utilization, and associated challenges at JMC, highlighting broader trends relevant to many LMICs. It reveals a high prevalence of broad-spectrum antibiotic use, particularly third-generation cephalosporins, along with notable variability in prescribing practices. The findings also point to limited diagnostic test utilization despite some of the patients (7/16, 43.8%) having positive culture results and gaps in adherence to treatment guidelines, underscoring the urgent need for comprehensive antimicrobial stewardship programs (ASPs) to curb the rising threat of AMR.

The study revealed a high rate of empiric antibiotic use, with 70.3% of patients receiving antibiotics for therapeutic reasons. This may be due to limited diagnostic resources or a precautionary approach taken by clinicians. While empiric antibiotic therapy is sometimes necessary, especially in severe or life-threatening conditions [22], this practice highlights the urgent need for rapid diagnostic tests to ensure optimal antibiotic use. Additionally, there was a concerning trend of prolonged antibiotic use for prophylactic purposes, with a median duration of 3 days (range: 1–17 days), despite the guidelines recommending discontinuation within 24 h if no infection is confirmed [23]. This prolonged use indicates a lack of clear distinction between therapeutic and prophylactic regimens, likely due to inconsistent clinical practices or inadequate protocols.

The predominant use of “Watch” group antibiotics, particularly ceftriaxone, reflects a global trend in LMICs where broad-spectrum agents are often over-prescribed due to ease of use, broad activity spectrum, high rate of resistant bacterial pathogens in the setting, and availability [1,3,8]. While these antibiotics offer coverage for a wide range of pathogens, their overuse significantly increases the risk of developing multidrug-resistant organisms (MDROs), such as extended-spectrum beta-lactamase-producing Enterobacterales and methicillin-resistant *Staphylococcus aureus* [6,7,24]. The study’s findings, where ceftriaxone accounted for 44.65 DOTs per 100 patient-days, underscore an urgent need to reassess the use of these agents.

The over-reliance on ceftriaxone is concerning, particularly because third-generation cephalosporins have been associated with the induction of beta-lactamase resistance mechanisms in several bacterial species [14]. Previous studies in Ethiopia have similarly reported high levels of ceftriaxone use, reflecting a broader national trend that is likely contributing to rising AMR rates [5,15,25]. This pattern of use suggests a need for more stringent guidelines and stricter adherence to protocols that limit the use of broad-spectrum antibiotics to cases where they are absolutely necessary.

Although our previous findings showed a high rate of multi-resistant bacterial pathogens [25,26,27], this study revealed limited use of culture and AST results, with only 4.2% of patients undergoing microbiological evaluation, and 43.8% (7/16) had positive culture results, despite the availability of these services at the hospital. Even when AST was performed, treatment adjustments based on the results were not observed. This highlights a significant gap in integrating diagnostic data into clinical decision-making, a problem that has been noted in other studies across similar settings [28,29].

The absence of routine microbiological testing prevents clinicians from accurately identifying pathogens and their resistance profiles, leading to empirical prescribing practices that may be inappropriate or suboptimal [16]. The reliance on empirical treatment, while necessary in certain contexts, can result in the overuse of broad-spectrum antibiotics, contributing to the selection pressure that drives AMR. Diagnostic stewardship, where clinicians are supported in ordering and interpreting diagnostic tests, is critical to improving the accuracy of antibiotic prescribing and should be integrated as a core component of ASPs [20]. Adequate antimicrobial stewardship requires a robust healthcare infrastructure that includes comprehensive surveillance systems to monitor antibiotic use and resistance patterns. Additionally, staff education is essential to ensure that healthcare providers understand the principles of responsible antibiotic prescribing and the impact of resistance. Implementing relevant methods, such as clinical guidelines and decision-support tools, can further enhance the effectiveness of stewardship programs, promoting optimal antibiotic use and improving patient outcomes [30,31].

The study found considerable variation in empirical antibiotic regimens across different wards for similar clinical indications, suggesting inconsistent adherence to local or international treatment guidelines. For instance, empirical regimens for conditions like community-acquired pneumonia and bacterial meningitis varied significantly, which could lead to inappropriate treatment and adverse patient outcomes [10,12].

This variation in prescribing practices is often driven by factors such as differences in clinician training, inconsistent access to essential antibiotics, and the absence of locally relevant guidelines based on antibiogram data. To address this, it is crucial to develop evidence-based, locally tailored treatment guidelines that reflect specific resistance patterns observed at JMC. Establishing a local antibiogram, which compiles and analyzes resistance data specific to the hospital’s patient population, can guide more targeted empiric therapy and help reduce the overuse of broad-spectrum antibiotics [32,33]. Countries like India and South Africa have successfully implemented various initiatives to improve antibiotic use and patient outcomes. These initiatives include raising awareness through campaigns, training healthcare providers, enhancing surveillance systems, improving infection prevention and control measures, and implementing an antibiotic stewardship program [34,35].

Therefore, the study underscores the importance of implementing comprehensive ASPs at JMC. To ensure the effective implementation of ASPs, it is crucial to enhance the surveillance system, educate staff, and establish effective infection prevention and control measures. A well-implemented ASP can help address these challenges by optimizing antibiotic use, reducing AMR, and improving patient outcomes [32]. It requires a multidisciplinary approach, including collaboration between clinicians, pharmacists, microbiologists, and hospital administrators. ASPs should focus on education, diagnostic stewardship, and the development of evidence-based guidelines informed by local antibiograms. The findings from a previous study by Gashaw et al. provide valuable insights for shaping local treatment protocols that can minimize the inappropriate use of broad-spectrum antibiotics [25]. However, implementing ASPs in resource-limited settings can be challenging due to constraints in infrastructure, laboratory capacity, and workforce [36].

The overuse of antibiotics and rising AMR rates at JMC reflect broader public health challenges faced by Ethiopia and many other LMICs. AMR threatens the sustainability of healthcare systems, leading to increased morbidity, mortality, and healthcare costs [37,38]. Without urgent and coordinated efforts to improve antibiotic prescribing practices, the effectiveness of available antibiotics will continue to decline, making it increasingly difficult to treat common infections.

Given the global nature of AMR, efforts to combat it at JMC will also contribute to broader regional and international strategies. Effective implementation of ASPs, guided by WHO recommendations, can serve as a model for other healthcare facilities in Ethiopia and similar settings, helping to curb the spread of AMR and preserving the efficacy of antibiotics for future generations [12,39].

### Strengths and Limitations

This study provides a comprehensive analysis of antibiotic prescribing patterns, diagnostic utilization, and treatment outcomes among patients admitted to a tertiary hospital in Ethiopia. However, it has several limitations worth mentioning. The reliance on data from a single tertiary care hospital limits the generalizability of findings to other settings, particularly primary care facilities where prescribing practices may differ. Additionally, the study’s observational design did not allow for a detailed assessment of the appropriateness of prescriptions based on clinical indications and patient outcomes. We also did not collect data on the clinicians’ reasoning behind their choice of antibiotics. Future research should aim to evaluate antibiotic prescribing practices across multiple healthcare settings in Ethiopia, assess the impact of implementing ASPs, and explore the barriers to effective diagnostic use.

## 5. Conclusions

This study highlights critical issues in antibiotic prescribing practices at JMC, including overuse of broad-spectrum “Watch” antibiotics, insufficient utilization of microbiological diagnostics, and inconsistent adherence to treatment guidelines. These practices contribute to the growing problem of AMR, threatening the effectiveness of available antibiotics and patient safety. To address these challenges, there is an urgent need to strengthen ASPs at JMC. Key recommendations include developing evidence-based local treatment guidelines informed by antibiogram data, improving access to and use of microbiological diagnostics, and implementing comprehensive ASPs. Through coordinated efforts, healthcare providers can optimize antibiotic use, reduce AMR, and improve patient outcomes, setting a precedent for similar facilities in Ethiopia and other LMICs.

## Figures and Tables

**Table 1:** Background characteristics of study participants.

Variables		Number	Percent
Sex	Female	177	46.1
	Male	207	53.9
Age	<2 months	38	9.9
	2 months–14 years	113	29.4
	15–49 years	180	46.9
	≥50 years	53	13.8
Residency	Urban	134	34.9
	Rural	250	65.1
Educational status	Cannot read and write	130	33.9
	No formal education but can read and write	91	23.7
	Elementary School	52	13.5
	High School	45	11.7
	Above high school	66	17.2
Occupation	Farmer	177	46.1
	Civil servant (public employee)	68	17.7
	Merchant	40	10.4
	Student	36	9.4
	Daily Laborer	23	6.0
	Others	40	10.4
Ward of admission	Pediatrics	96	25.5
	Emergency room	60	15.6
	Obstetrics and Gynecology	49	12.8
	General surgery	41	10.7
	NICU	33	8.6
	Internal Medicine	25	6.5
	Orthopedic	23	6.0
	Ophthalmology	23	6.0
	Oral and maxillofacial surgery	19	4.9
	ICU	15	3.9
Underlying medical conditions	Diabetes mellitus	12	3.1
	Hypertension	12	3.1
	Heart failure	6	1.6
	HIV infection	4	1.0
	Tuberculosis	3	0.8
	Severe malnutrition	3	0.8
	Others	4	1.0
Main reasons for admission	Elective surgery	73	19.0
	Abscess and skin/soft tissue infection	38	9.9
	Complicated labor	32	8.3
	Community-acquired pneumonia	32	8.3
	Sepsis/septic shock	31	8.1
	Bacterial meningitis	24	6.3
	Eye disorders (including infections)	23	6.0
	Acute abdomen	20	5.2
	Heart failure	18	4.7
	Trauma/injury	15	3.9
	Diabetes mellitus/Hypertension/Stroke	14	3.6
	Hyaline membrane diseases	9	2.3
	Acute renal insufficiency	8	2.1
	HIV/TB/Malaria	9	2.3
	Airway obstruction (COPD/UAO)	6	1.6
	Others ^i^	32	8.3

HIV: human immunodeficiency virus; ICU: intensive care unit; NGO: nongovernmental organization; NICU: neonatal intensive care unit; UAO: upper airway obstruction; Others ^i^: Unilateral Vocal Cord Paralysis (UVP) (5), surgical site infection (SSI) (5), osteomyelitis (4), acute gastroenteritis (AGE) (3), pertussis (3), urinary tract infection (UTI) (3), Guillain–Barré Syndrome (2), neutropenic fever (2), Amoebiasis (1), and severe malnutrition (1).

**Table 2:** Indications for antibiotics.

Indications	Number	Percent
Therapeutic	270	70.3
Pneumonia	62	16.1
Abscess and skin/soft tissue infection	38	9.9
Sepsis/septic shock	32	8.3
Bacterial meningitis	27	7.0
Eye infection	18	4.7
Perforated viscus/abdominal surgery/intestinal obstruction	19	4.9
Urinary tract infection	13	3.4
Trauma/injury	11	2.9
Hyaline membrane disease (HMD)	9	2.3
DM/heart failure/stroke	9	2.3
Acute gastroenteritis (AGE)	7	1.8
Others	25	6.5
Prophylactic	114	29.7
Surgical prophylaxis	95	24.7
Complicated labor (prevention of peripartum infection)	14	3.6
Rheumatic heart disease prophylaxis	5	1.3

Others: nephrolithiasis (1), acute glomerulonephritis (4), anemia (1), cholecystitis (1), chorioamnionitis (3), empyema (2), heart failure (4), malignancy (1), neutropenic fever (2), osteomyelitis (4), pertussis (1), tuberculosis (1).

**Table 3:** Antibiotic consumption pattern and route of administration.

Antibiotic Class	Antimicrobial Agents	Route	Frequency of Regimen N (%)	DOTs Cumulative	DOTs/100 Patient Days
Penicillin	Ampicillin	IV	54 (8.5)	235	8.16
	Amoxicillin-clavulanic acid	PO	23 (3.6)	34	1.18
	Cloxacillin	IV	21 (3.3)	100	3.47
	Amoxicillin	PO	12 (1.9)	35	1.22
	Penicillin	IV/IM	7 (1.1)	14	0.49
Macrolide	Azithromycin	PO	22 (3.5)	107	3.72
	Clarithromycin	PO	1 (0.2)	6	0.21
Cephalosporins	Ceftriaxone	IV	268 (42.3)	1286	44.65
	Cefalexin	PO	4 (0.4)	14	0.49
	Ceftazidime	IV	1 (0.2)	3	0.10
Amphenicols	Chloramphenicol	IV	20 (3.2)	54	1.88
Quinolones/fluoroquinolones	Ciprofloxacin	IV/PO	23 (3.6)	126	4.38
	Norfloxacin	PO	3 (0.5)	10	0.35
Sulfonamides + Diaminopyrimidines	Trimethoprim-sulfamethoxazole	PO	2 (0.3)	7	0.24
Tetracyclines	Doxycycline	PO/IV	9 (1.4)	28	0.97
Aminoglycosides	Gentamicin	IV	68 (10.7)	313	10.87
Nitroimidazoles	Metronidazole	IV/PO	86 (13.6)	480	16.67
Glycopeptides	Vancomycin	IV	10 (1.6)	28	0.97

DOTs—days of therapy; IM—intramuscular; IV—intravenous; PO—oral.

**Table 4:** Empiric antibiotic regimens used for common indications.

Indications	Antibiotics Regimen Used	N (%)	STG Recommendation
Neonatal sepsis (*n* = 32)	Ampicillin + gentamicin	22 (68.7)	Ampicillin ^†^ (or Benzylpenicillin) PLUS Gentamicin ^†^
	Ceftriaxone + gentamicin	4 (12.5)	
	Ceftriaxone	2 (6.3)	
	Ceftriaxone + metronidazole	2 (6.3)	
	Ampicillin + gentamicin + cloxacillin	2 (6.3)	
Community-acquired severe pneumonia in adults (*n* = 34)	Ceftriaxone	17 (28.3)	Ceftriaxone ^†^ (or Benzyl penicillin) PLUS Azithromycin ^†^ (or clarithromycin)
	Ceftriaxone + azithromycin	15 (25.0)	
	Ceftriaxone + doxycycline	2 (3.2)	
Community-acquired bacterial meningitis (*n* = 27)	Ceftriaxone	9 (33.3)	Ceftriaxone ^†^ PLUS Vancomycin ^†^ Alternative Benzyl penicillin PLUS chloramphenicol Add ampicillin in high risk for *Listeria monocytogenes*
	Ceftriaxone + vancomycin	3 (11.1)	
	Ceftriaxone + metronidazole	3 (11.1)	
	Ampicillin + gentamicin	3 (11.1)	
	Ampicillin + ceftriaxone + gentamicin	1 (3.7)	
	Ampicillin + gentamicin + cloxacillin	1 (3.7)	
	Ceftriaxone + doxycycline	2 (7.4)	
	Ceftriaxone + metronidazole + doxycycline	1 (3.7)	
	Ceftriaxone + metronidazole	4 (14.9)	
Community-acquired urinary tract infection (*n* = 13)	Ceftriaxone	5 (38.4)	Ciprofloxacin ^†^ or Norfloxacin^†^ Alternative Trimethoprim-sulphamethoxazole OR Nitrofurantoin OR Ceftriaxone (for acute severe infection)
	Ciprofloxacin	2 (15.4)	
	Ceftriaxone + metronidazole	1 (7.7)	
	Cloxacillin	1 (7.7)	
	Cephalexin	2 (15.4)	
	Ceftriaxone + ciprofloxacin	2 (15.4)	
Skin/soft tissue infections (excluding surgical site infection) (*n* = 33)	Ceftriaxone + cloxacillin	7 (21.2)	Furuncle, carbuncle, folliculitis, impetigo Cloxacillin ^†^ Alternative (Erythromycin or cephalexin) Cellulitis and Erysipelas Procaine Penicillin ^†^ Alternative Cloxacillin OR Erythromycin OR Cephalexin
	Ceftriaxone	6 (18.2)	
	Amoxicillin/clavulanic acid	6 (18.2)	
	Ceftriaxone + metronidazole + chloramphenicol	4 (12.1)	
	Ampicillin + chloramphenicol	3 (9.1)	
	Cloxacillin + chloramphenicol	3 (9.1)	
	Ceftriaxone + metronidazole + chloramphenicol	2 (6.1)	
	Ceftriaxone + metronidazole	1 (3.0)	
	Cloxacillin	1 (3.0)	
Perioperative prophylaxis (*n* = 94)	Ceftriaxone	65 (68.4)	Cefazolin ^†^, unless specified
	Ceftriaxone + metronidazole	22 (23.2)	
	Ceftriaxone + gentamicin	3 (3.2)	
	Ciprofloxacin	2 (2.1)	
	Ampicillin	1 (1.1)	
	Ampicillin + chloramphenicol	1 (1.1)	
	Ceftriaxone + cloxacillin	1 (1.1)	

STG: standard treatment guideline; ^†^: first-line antibiotic.

## Data Availability

The datasets used in the current study are available from the corresponding author on reasonable request.

## Abbreviations

AMR	Antimicrobial resistance
ASP	Antimicrobial stewardship program
AST	Antimicrobial susceptibility test
AWaRe	Access, Watch, and Reserve
DOTs	Days of therapy
LMICs	Low- and middle-income countries
MDROs	Multidrug-resistant organisms
WHO	World Health Organization
